# Antioxidant Properties of Fucoidan Alleviate Acceleration and Exacerbation of Hippocampal Neuronal Death Following Transient Global Cerebral Ischemia in High-Fat Diet-Induced Obese Gerbils

**DOI:** 10.3390/ijms20030554

**Published:** 2019-01-28

**Authors:** Ji Hyeon Ahn, Myoung Cheol Shin, Dae Won Kim, Hyunjung Kim, Minah Song, Tae-Kyeong Lee, Jae-Chul Lee, Hyeyoung Kim, Jun Hwi Cho, Young-Myeong Kim, Jong-Dai Kim, Soo Young Choi, Moo-Ho Won, Joon Ha Park

**Affiliations:** 1 Department of Biomedical Science and Research Institute for Bioscience and Biotechnology, Hallym University, Chuncheon, Gangwon 24252, Korea; nqubik@hanmail.net (J.H.A.); sychoi@hallym.ac.kr (S.Y.C.); 2 Department of Emergency Medicine, Kangwon National University Hospital, School of Medicine, Kangwon National University, Chuncheon, Gangwon 24341, Korea; dr10126@naver.com (M.C.S.); hae1127@hanmail.net (H.K.); cjhemd@kangwon.ac.kr (J.H.C.); 3 Department of Biochemistry and Molecular Biology, and Research Institute of Oral Sciences, College of Dentistry, Gangnung-Wonju National University, Gangneung, Gangwon 25457, Korea; kimdw@gwnu.ac.kr; 4 Department of Neurobiology, School of Medicine, Kangwon National University, Chuncheon, Gangwon 24341, Korea; nicolehkim@naver.com (H.K.); zlscydn@naver.com (M.S.); xorud312@naver.com (T.-K.L.); anajclee@kangwon.ac.kr (J.-C.L.); 5 Department of Anesthesiology and Pain Medicine, Chungju Hospital, Konkuk University School of Medicine, Chungju, Chungcheongbuk 27376, Korea; 6 Department of Molecular and Cellular Biochemistry, School of Medicine, Kangwon National University, Chuncheon, Gangwon 24341, Korea; ymkim@kangwon.ac.kr; 7 Division of Food Biotechnology, School of Biotechnology, Kangwon National University, Chuncheon, Gangwon 24341, Korea; jongdai@kangwon.ac.kr

**Keywords:** obesity, transient global cerebral ischemia, fucoidan, neuroprotection, oxidative stress, antioxidant enzymes

## Abstract

Fucoidan, a natural sulfated polysaccharide, displays various biological activities including antioxidant properties. We examined the neuroprotective effect of fucoidan against transient global cerebral ischemia (tGCI) in high-fat diet (HFD)-induced obese gerbils and its related mechanisms. Gerbils received HFD for 12 weeks and fucoidan (50 mg/kg) daily for the last 5 days during HFD exposure, and they were subjected to 5-min tGCI. Pyramidal cell death was observed only in the CA 1 area (CA1) of the hippocampus in non-obese gerbils 5 days after tGCI. However, in obese gerbils, pyramidal cell death in the CA1 and CA2/3 occurred at 2 days and 5 days, respectively, after tGCI. In the obese gerbils, oxidative stress indicators (dihydroethidium, 8-hydroxyguanine and 4-hydroxy-2-nonenal) were significantly enhanced and antioxidant enzymes (SOD1 and SOD2) were significantly reduced in pre- and post-ischemic phases compared to the non-obese gerbils. Fucoidan treatment attenuated acceleration and exacerbation of tGCI-induced neuronal death in the CA1–3, showing that oxidative stress was significantly reduced, and antioxidant enzymes were significantly increased in pre- and post-ischemic phases. These findings indicate that pretreated fucoidan can relieve the acceleration and exacerbation of ischemic brain injury in an obese state via the attenuation of obesity-induced severe oxidative damage.

## 1. Introduction

Transient global cerebral ischemia (tGCI) occurs commonly during cardiac arrest or resuscitation, which hinders the supply of blood to the brain and causes the development of irreversible brain damage [1,2]. An important feature of tGCI-induced injury is “delayed neuronal death (DND)” in the cornu ammonis 1 (CA1) area of the hippocampus: the DND occurs selectively in pyramidal neurons of the CA1 area at several days after tGCI [3,4]. Until now, diverse mechanisms of tGCI-induced injury have been suggested by many researchers [5]. Among the mechanisms, oxidative stress via excessive reactive oxygen species (ROS) production following tGCI has been considered as a major contributor to tGCI-induced DND [6,7]. Thus, antioxidant therapy, which can effectively inhibit excessive production of ROS through the activation of endogenous antioxidant enzymes, is considered to be a potential therapeutic approach for tGCI-induced injury [8,9].

Obesity, a condition characterized by immoderate fat accumulation in the body, is a well-established risk factor of cerebral ischemia [10]. Obesity is known to cause the increase of oxidative stress in the brain [11]. Patients with obesity have a higher mortality rate than non-obese patients after ischemic insults [12,13]. In animal studies, obesity induced by high-fat diet (HFD) results in exacerbated ischemic brain injury [14,15,16]. Thus, we must investigate protective strategies against ischemic brain injury in obesity.

Medicinal plants and natural substances isolated from them have been thought to be ideal candidate agents for the effective prevention and treatment of brain ischemia because they possess various pharmacological activities in the treatment of human ailments [17,18]. Fucoidan is a natural sulfated polysaccharide found mainly in cell-wall matrix of various brown algae species and displays pleiotropic activities, including antioxidant, anti-inflammatory, and anti-thrombotic properties [19,20,21]. Many studies have demonstrated that fucoidan has strong neuroprotective effects against ischemic brain injury in non-obese animal models. For instance, Che et al. have showed that pretreated fucoidan significantly reduces brain infarct size after transient focal cerebral ischemia in rats [22], and Kim et al. have reported that pretreated fucoidan attenuates tGCI-induced neuronal death in the hippocampal CA1 area in gerbils [23]. Furthermore, pretreatment with fucoidan effectively inhibits lipopolysaccharide-induced exacerbation of transient focal cerebral ischemic injury in rats [24]. In addition, recent studies have shown that post-treatment with fucoidan attenuates hindlimb ischemic injury in mouse and rat models of type 2 diabetes [18,25]. To the best of our knowledge, however, no studies on beneficial effects of fucoidan against ischemic brain injury in animal models of obesity have been reported. Therefore, the aim of this study was to investigate the neuroprotective effect of fucoidan against tGCI-induced injury and its mechanism in HFD-induced obese gerbils, which have been used as a useful animal model for studying the metabolic abnormalities associated with obesity and the mechanisms of tGCI-induced neuronal death, and as a good screening animal for assessing neuroprotective agents [26,27]. It has been well established that the gerbil lacks posterior communicating arteries that connect the carotid and vertebrobasilar arterial system, unlike the mouse and rat. Accordingly, tGCI is easily and reproducibly induced by a simple bilateral common carotid artery occlusion [28,29].

## 2. Results

### 2.1. Physiological Characteristics

Body weight, blood glucose, serum triglyceride, and total cholesterol levels in the HFD-fed gerbils were significantly higher than those in the normal diet (ND)-fed gerbils. In the HFD/fucoidan-fed gerbils, significant changes in physiological characteristics were not observed compared to those in the HFD-fed gerbils (Table 1). This result indicates that pretreated fucoidan in the HFD-fed obese gerbils does not affect changes in the physiological characteristics induced by HFD.

### 2.2. Neuronal Death in HFD-Ischemia Group

**Figure 1:**
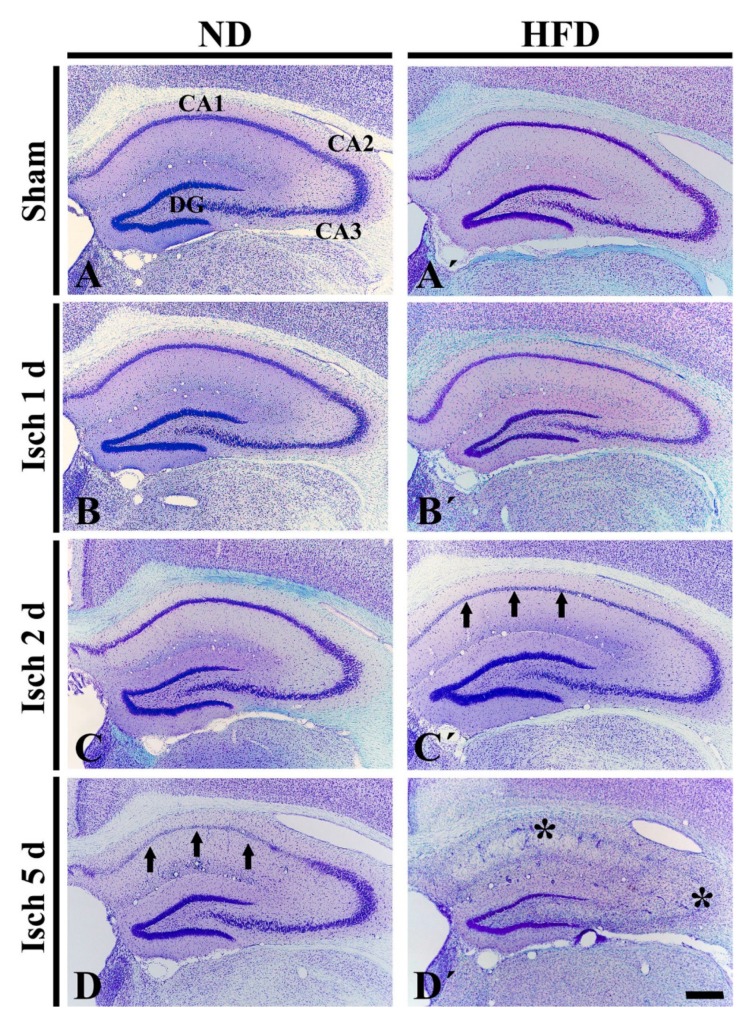
CV staining in the hippocampus of the ND-fed (left column) and HFD-fed (right column) groups at sham (**A**,**A**’), 1 day (**B**,**B**′), 2 days (**C**,**C**′) and 5 days (**D**,**D**′) after tGCI. In the ND-ischemia group, CV^+^ cells are pale (damaged) in the CA1 area (arrows) 5 days after tGCI. In the HFD-ischemia-group, damaged CV^+^ cells in the CA1 area (arrows) is shown 2 days after tGCI, and, 5 days after tGCI, severely damaged CV^+^ cells are shown in the CA2/3 area (asterisk) as well as CA1 area (asterisk). DG, dentate gyrus. Scale bar = 400 μm.

As shown in Figure 1A, CV^+^ cells were easily identified in all layers of the hippocampus of the ND-sham group. In the ND-ischemia group, a significant change in CV^+^ cells was not observed until 2 days after tGCI (Figure 1B,C). However, at 5 days after tGCI, CV^+^ cells of the stratum pyramidale only in the CA1 area were significantly pale, which indicates cell damage (Figure 1D). In the HFD-sham group, the distribution pattern of the CV^+^ cells was similar to that in the ND-sham group (Figure 1A′). In the HFD-ischemia group, CV^+^ cells of the stratum pyramidale in the CA1 area were damaged 2 days after tGCI (Figure 1B′,C′), and, at 5 days after tGCI, CV^+^ cells were severely damaged in the CA2/3 area as well as in the CA1 area (Figure 1D′). This finding demonstrates that HFD-induced obesity results in the acceleration and exacerbation of tGCI-induced injury in the hippocampus.

### 2.3. Neuroprotection by Fucoidan in HFD-Ischemia Group

#### 2.3.1. NeuN^+^ Cells

**Figure 2:**
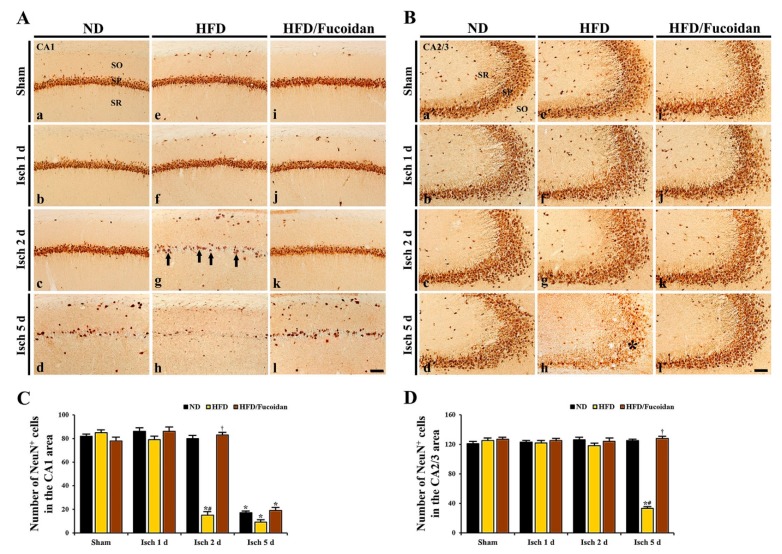
(**A**,**B**) NeuN immunohistochemistry in the CA1 (**A**) and CA2/3 (**B**) areas of the ND-fed (left columns), HFD-fed (middle columns) and HFD/Fucoidan-fed (right columns) groups at sham (a, e,i,a′,e′,i′), 1 day (b,f,j,b′,f′,j′), 2 days (c,g,k,c′,g′,k′) and 5 days (d,h,l,d′,h′,l′) after tGCI. NeuN^+^ CA1 pyramidal cells (arrows) in the HFD-ischemia group are earlier lost (2 days after tGCI); however, NeuN^+^ CA1 pyramidal cells in the HFD/Fucoidan-ischemia group are shown at this time. In the CA2/3 area, NeuN^+^ pyramidal cells (asterisk) are decreased 5 days after tGCI; however, NeuN^+^ pyramidal cells in the HFD/Fucoidan-ischemia group are not lost. SO, stratum oriens; SP, stratum pyramidale; SR, stratum radiatum. Scale bar = 60 μm. (**C** and **D**) The mean number of NeuN^+^ pyramidal cells in the CA1 (**C**) and CA2/3 (**D**) areas (*n* = 7/group). The bars indicate the means ± SEM. * *P* < 0.05, vs. each sham group, ^#^ *P* < 0.05 vs. ND-fed group, ^†^ *P* < 0.05 vs. HFD-fed group.

**CA1 area:** Cells in the stratum pyramidale of the CA1 area, which are called CA1 pyramidal cells or neurons, were well immunostained with NeuN in the ND-sham group (Figure 2Aa). In the HFD- and HFD/Fucoidan-sham groups, NeuN^+^ CA1 pyramidal cells were no different from those in the ND-sham group (Figure 2Ae,Ai,C). In the ND-ischemia group, NeuN^+^ CA1 pyramidal cells were not significantly altered until 2 days after tGCI (Figure 2Ab,Ac); however, a significant loss of NeuN^+^ CA1 pyramidal cells was observed 5 days after tGCI (Figure 2Ad): the mean number of the NeuN^+^ pyramidal cells was 82.3 ± 2.2 cells/250 × 250 μm (Figure 2C). In the HFD-ischemia group, a significant loss of NeuN^+^ CA1 pyramidal cells was observed 2 days after tGCI (Figure 2Af,Ag,C). Five days after tGCI, NeuN^+^ CA1 pyramidal cells were further decreased (9.3 ± 2.4 cells/250 × 250 μm) (Figure 2Ah, C). In the HFD/Fucoidan-ischemia group, the distribution pattern and numbers of NeuN^+^ CA1 pyramidal cells were similar to those in the ND-ischemia group, namely, a loss of NeuN^+^ CA1 pyramidal cells was observed only at 5 days after tGCI (Figure 2Aj–Al,C).

**CA2/3 area:** In all sham groups, CA2/3 pyramidal cells were also immunostained with NeuN, and no significant differences in their distribution and numbers were found between all sham groups (Figure 2Ba′,Be′,Bi′,D). In the ND-ischemia group, any changes in NeuN^+^ CA2/3 pyramidal cells were not observed until 5 days after tGCI (Figure 2Bb′–Bd′,D). In the HFD-ischemia group, NeuN^+^ CA2/3 pyramidal cells were not changed until 2 days after tGCI (Figure 2Bf′,Bg′,D). However, at 5 days after tGCI, a significant loss of NeuN^+^ CA2/3 pyramidal cells was found (Figure 2Bh′), showing that the mean number of the pyramidal cells was 31.4 ± 2.5 cells/250 × 250 μm (Figure 2D). In the HFD/Fucoidan-ischemia group, the distribution pattern and numbers of NeuN^+^ CA2/3 pyramidal cells were not significantly altered until 5 days after tGCI compared to those in the HFD/Fucoidan-sham group (Figure 2Bj′–Bl′,D).

This finding indicates that pretreated fucoidan attenuates the acceleration and exacerbation of tGCI-induced damage/death of CA1 and CA2/3 pyramidal cells under obese state.

#### 2.3.2. F-J B^+^ Cells

**Figure 3:**
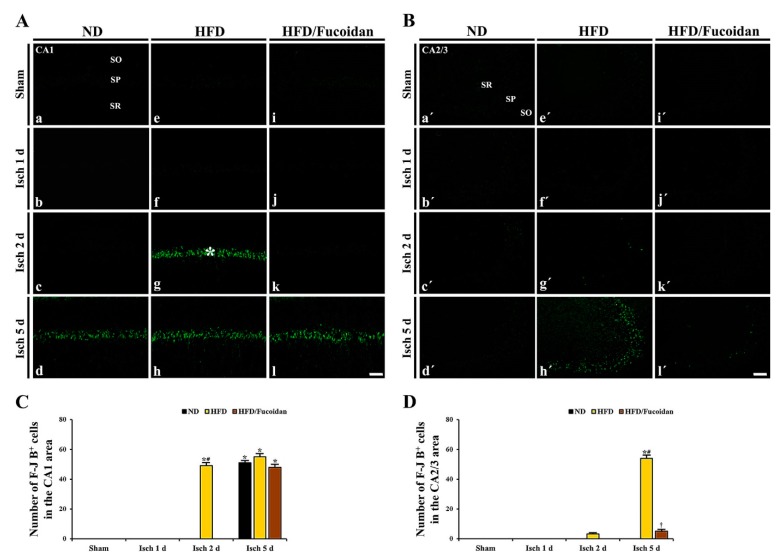
(**A**,**B**) F-J B histofluorescence staining in the CA1 (**A**) and CA2/3 (**B**) areas of the ND-fed (left columns), HFD-fed (middle columns) and HFD/Fucoidan-fed (right columns) groups at sham (a, e i, a′, e′, i′), 1 day (b, f, j, b′, f′, j′), 2 days (c, g, k, c′, g′, k′) and 5 days (d, h, l, d′, h′, l′) after tGCI. In the HFD-ischemia group, many F-J B ^+^ CA1 pyramidal cells (asterisk) are earlier shown (2 days after tGCI). In the HFD/Fucoidan-ischemia group, F-J B ^+^ CA1 pyramidal cells are not observed 2 days after tGCI. Note that F-J B ^+^ CA2/3 pyramidal cells are shown only in the HFD-ischemia group 5 days after tGCI. Scale bar = 60 μm. (**C**,**D**) The mean number of F-J B^+^ cells in the CA1 (**C**) and CA2/3 (**D**) areas (*n* = 7/group). The bars indicate the means ± SEM. * *P* < 0.05, vs. each sham group, ^#^ *P* < 0.05 vs. ND-fed group, ^†^ *P* < 0.05 vs. HFD-fed group.

**CA1 area:** In all sham groups, no F-J B^+^ cells were found in the CA1 area (Figure 3Aa, Ae, Ai). In the ND-ischemia group, F-J B^+^ cells were not observed until 2 days after tGCI (Figure 3Ab, Ac); however, at 5 days after tGCI, many F-J B^+^ CA1 pyramidal cells were detected (51.3 ± 1.4 cells/250 X 250 μm) (Figure 3Ad,C). In the HFD-ischemia group, abundant F-J B^+^ CA1 pyramidal cells were detected 2 days after tGCI, and more F-J B^+^ CA1 pyramidal cells (55.3 ± 2.2 cells/250 × 250 μm) were shown 5 days after tGCI (Figure 3Af–Ah, C). In the HFD/Fucoidan-ischemia group, no F-J B^+^ CA1 pyramidal cells were detected at 2 days after tGCI; however, many F-J B^+^ CA1 pyramidal cells (48.6 ± 1.9 cells/250 X 250 μm) were shown 5 days after tGCI (Figure 3Aj–Al, C).

**CA2/3 area:** F-J B^+^ CA2/3 pyramidal cells were not detected in all sham groups (Figure 3Ba′, Be′, Bi′). In the ND-ischemia group, no F-J B^+^ CA2/3 pyramidal cells were observed until 5 days after tGCI (Figure 3Bd′–Bd′). In the HFD-ischemia group, some F-J B^+^ CA2/3 pyramidal cells (3.3 ± 0.5 cells/250 × 250 μm) were observed 2 days after tGCI, and, at 5 days after tGCI, many F-J B^+^ CA2/3 pyramidal cells (54.8 ± 2.4 cells/250 X 250 μm) were shown (Figure 3Bf′-Bh′, D). In the HFD/Fucoidan-ischemia group, no F-J B^+^ CA2/3 pyramidal cells were detected 2 days after tGCI (Figure 3Bj′, Bk′), and a few F-J B^+^ CA2/3 pyramidal cells (5.2 ± 1.2 cells/250 X 250 μm) were detected 5 days after tGCI (Figure 3Bl′ and D).

This result demonstrates that pretreated fucoidan protects CA1 and CA2/3 pyramidal cells from obesity-induced augmentation of ischemic brain injury.

### 2.4. Inhibitory Effect of Fucoidan on Oxidative Stress in HFD-Ischemia Group

#### 2.4.1. DHE Fluorescence

**Figure 4:**
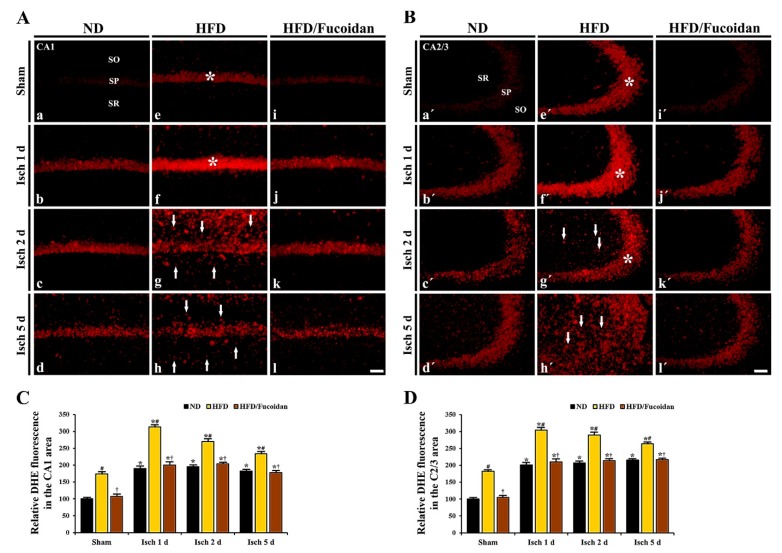
(**A**,**B**) DHE fluorescence staining in the CA1 (**A**) and CA2/3 (**B**) areas of the ND-fed (left columns), HFD-fed (middle columns) and HFD/Fucoidan-fed (right columns) groups at sham (a, e i, a′, e′, i′), 1 day (b, f, j, b′, f′, j′), 2 days (c, g, k, c′, g′, k′) and 5 days (d, h, l, d′, h′, l′) after tGCI. In the HFD-sham and ischemia groups, DHE fluorescence is significantly increased in pyramidal cells (asterisks) and newly expressed in non-pyramidal cells (arrows) of the CA1-3 areas; however, DHE fluorescence in the HFD/Fucoidan-sham and ischemia groups is significantly decreased. Scale bar = 60 μm. (**C**,**D**) Quantitative analysis of DHE fluorescence in the CA1 (**C**) and CA2/3 (**D**) areas (*n* = 7/group). The bars indicate the means ± SEM. * *P* < 0.05 vs. each sham group, ^#^ *P* < 0.05 vs. ND-fed group, ^†^ *P* < 0.05 vs. HFD-fed group.

**CA1 area:** DHE fluorescence to detect superoxide anions was very weakly shown in CA1 pyramidal cells in the ND-sham group (Figure 4Aa). In the ND-ischemia group, DHE fluorescence was significantly increased (about 191% of ND-sham group) in CA1 pyramidal cells 1 day after tGCI (Figure 4Ab, C). The increased DHE fluorescence was maintained until 5 days after tGCI (Figure 4Ac, Ad, C). In particular, at 5 days after tGCI, strong DHE fluorescence was newly shown in non-pyramidal cells of the stratum oriens and radiatum (Figure 4Ad). In the HFD-sham group, DHE fluorescence in CA1 pyramidal cells was significantly higher (about 175% of ND-sham group) than that in the ND-sham group (Figure 4Ae, C). In the HFD-ischemia group, DHE fluorescence in CA1 pyramidal cells was significantly increased (about 180% of HFD-sham group) 1 day after tGCI (Figure 4Af, C). Thereafter, DHE fluorescence in CA1 pyramidal cells was gradually decreased with a time-dependent manner; however, strong DHE fluorescence was shown in non-pyramidal cells at 2 days after tGCI, showing that the numbers of non-pyramidal cells with DHE fluorescence were decreased at 5 days after tGCI (Figure 4Ag, Ah). In the HFD/Fucoidan-sham group, DHE fluorescence in CA1 pyramidal cells was significantly lower (about 62% of HFD-sham group) compared to the HFD-sham group (Figure 4Ai, C). In the HFD/Fucoidan-ischemia group, DHE fluorescence in CA pyramidal cells was increased and maintained until 5 days after tGCI, showing that the increased DHE fluorescence was significantly lower than that in the HFD-ischemia group (Figure 4Aj–Al, C).

**CA2/3 area:** In the ND-sham group, the DHE fluorescence in CA2/3 pyramidal cells was similar to that in CA1 pyramidal cells (Figure 4Ba′). In the ND-ischemia group, the change pattern of DHE fluorescence in CA2/3 pyramidal cells was similar to that in the CA1 area of the ND-ischemia group (Figure 4Bd′, Bc′, Bd′, D). In the HFD-sham group, DHE fluorescence in CA2/3 pyramidal cells was also higher than the ND-sham group (Figure 4Be′, D). Also, the change pattern of DHE fluorescence in CA2/3 pyramidal cells of the HFD-ischemia group was similar to that in CA1 pyramidal cells (Figure 4Bf′, Bg′, Bh′, D). However, the change pattern of DHE fluorescence in non-pyramidal cells was different from that in CA1 pyramidal cells: strong DHE fluorescence was shown in many non-pyramidal cells at 5 days, not 2 days, after tGCI (Figure 4Bg′, Bh′). In the HFD/Fucoidan-sham and ischemia groups, DHE fluorescence in CA2/3 pyramidal cells was changed like that in CA1 pyramidal cells (Figure 4Aj′–Al′, D).

This finding indicates that pretreated fucoidan reduces obesity-induced severe increase in superoxide anions generation (DHE) in the CA1 and CA2/3 areas in pre- and post-ischemic phases.

#### 2.4.2. 8-OHG Immunoreactivity

**Figure 5:**
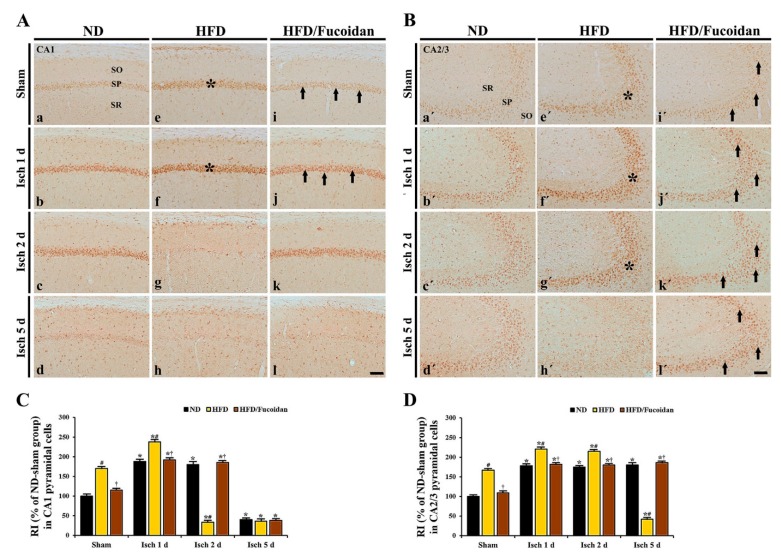
(**A**,**B**) 8-OHG immunohistochemistry in the CA1 (**A**) and CA2/3 (**B**) areas of the ND-fed (left columns), HFD-fed (middle columns) and HFD/Fucoidan-fed (right columns) groups at sham (a, e i, a′, e′, i′), 1 day (b, f, j, b′, f′, j′), 2 days (c, g, k, c′, g′, k′) and 5 days (d, h, l, d′, h′, l′) after tGCI. 8-OHG immunoreactivity in CA1-3 pyramidal cells (asterisks) of the HFD-sham and ischemia groups is significantly higher than hat in the ND-sham and ischemia groups; however, in the HFD/Fucoidan-sham and ischemia groups, 8-OHG immunoreactivity (arrows) is significantly lower than that in the HFD-sham and ischemia groups. Scale bar = 60 μm. (**C**,**D**) Quantitative analysis of 8-OHG immunoreactivity in CA1 (**C**) and CA2/3 (**D**) pyramidal cells (*n* = 7/group). The bars indicate the means ± SEM. * *P* < 0.05 vs. each sham group, ^#^ *P* < 0.05 vs. ND-fed group, ^†^ *P* < 0.05 vs. HFD-fed group.

**CA1 area:** Weak 8-OHG immunoreactivity was detected in CA1 pyramidal cells in the ND-sham group (Figure 4Aa). In the ND-ischemia group, 8-OHG immunoreactivity in CA1 pyramidal cells was about 189% and 181% of the ND-sham group, respectively, at 1 day and 2 days after tGCI (Figure 5Ab, Ac, C) and hardly shows 5 days after tGCI, because of the death of the CA1 pyramidal cells (Figure 5Ad, C). In the HFD-sham group, 8-OHG immunoreactivity in CA1 pyramidal cells was significantly higher (about 171% of the ND-sham group) than that in the ND-sham group (Figure 5Ae, C). In the HFD-ischemia group, 8-OHG immunoreactivity in CA1 pyramidal cells was increased by about 40% at 1 day after tGCI compared to the HFD-sham group; however, its immunoreactivity was hardly observed in CA1 pyramidal cells at 2 days and 5 days after tGCI due to the death of the CA1 pyramidal cells (Figure 5Af–Ah, C). In the HFD/Fucoidan-sham group, 8-OHG immunoreactivity in CA1 pyramidal cells was about 68% of the HFD-sham group (Figure 5Ai, C). In the HFD/Fucoidan-ischemia group, 8-OHG immunoreactivity in CA1 pyramidal cells was increased, but the immunoreactivity was about 80% of the HFD-ischemia group (Figure 5Aj, C). At 2 days after tGCI, 8-OHG immunoreactivity in CA1 pyramidal cells was not altered; however, at 5 days after tGCI, 8-OHG immunoreactivity was hardly shown due to the death of CA1 pyramidal cells (Figure 5Ak, Al, C).

**CA2/3 area:** In the ND-sham group, weak 8-OHG immunoreactivity was also observed in CA2/3 pyramidal cells (Figure 5Ba′). In the ND-ischemia group, 8-OHG immunoreactivity was about 179% of the ND-sham group at 1 day after tGCI, and the increased 8-OHG immunoreactivity was not changed until 5 days after tGCI (Figure 5Bb′–Bd′, D). In the HFD-sham group, 8-OHG immunoreactivity in CA2/3 pyramidal cells was also higher (about 167% of the ND-sham group) than that in the ND-sham group (Figure 5Be′, D). In the HFD-ischemia group, 8-OHG immunoreactivity was significantly increased (about 132% and 129%, respectively, of the HFD-sham group) at 1 day and 2 days after tGCI; however, at 5 days after tGCI, 8-OHG immunoreactivity in CA2/3 pyramidal cells was very low due to the death of CA2/3 pyramidal cells (Figure 5Bf′–Bh′, D). In the HFD/Fucoidan-sham and ischemia groups, the pattern of 8-OHG immunoreactivity in CA2/3 pyramidal cells was similar to that in the ND-sham and ischemia groups (Figure 5Bi′–Bl′, D).

This finding indicates that pretreated fucoidan attenuates severe oxidative RNA damage (8-OHG) induced by obesity in CA1 and CA2/3 pyramidal cells in pre- and post-ischemic phases.

#### 2.4.3. 4-HNE Immunoreactivity and Protein Level

**Figure 6:**
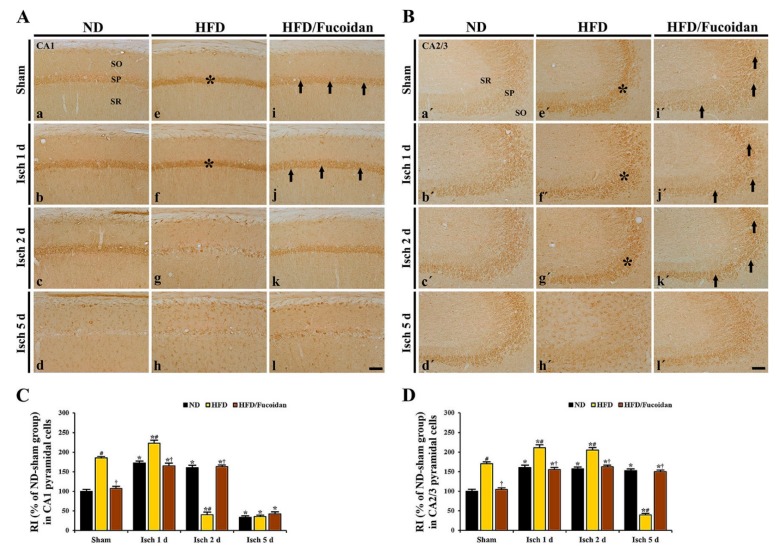
(**A**,**B**) 4-HNE immunohistochemistry in the CA1 (**A**) and CA2/3 (**B**) areas of the ND-fed (left columns), HFD-fed (middle columns) and HFD/Fucoidan-fed (right columns) groups at sham (a, e i, a′, e′, i′), 1 day (b, f, j, b′, f′, j′), 2 days (c, g, k, c′, g′, k′) and 5 days (d, h, l, d′, h′, l′) after tGCI. Asterisks show that 4-HNE immunoreactivity in CA1-3 pyramidal cells is significantly increased in the HFD-sham and ischemia groups. Arrows indicate that 4-HNE immunoreactivity in CA1-3 pyramidal cells is deceased in the HFD/Fucoidan-fed group compared to the HFD-fed group. Scale bar = 60 μm. (**C**,**D**) Quantitative analysis of 4-HNE immunoreactivity in the CA1 (**C**) and CA2/3 (**D**) pyramidal cells (*n* = 7/group). The bars indicate the means ± SEM. * *P* < 0.05 vs. each sham group, ^#^ *P* < 0.05 vs. ND-fed group, ^†^ *P* < 0.05 vs. HFD-fed group.

**Figure 7:**
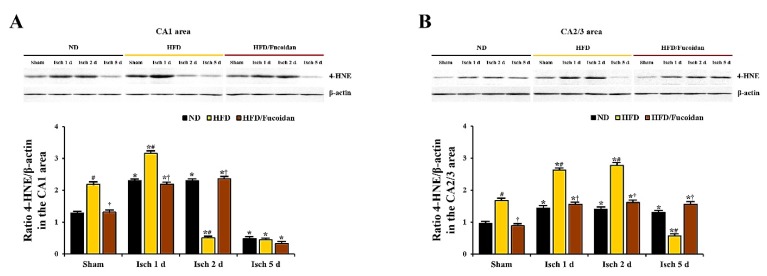
Western blot analysis of 4-HNE in the CA1 (**A**) and CA2/3 (**B**) areas (*n* = 6/group). Protein expression is normalized to β-actin. The bars indicate the means ± SEM. * *P* < 0.05 vs. each sham group, ^#^ *P* < 0.05 vs. ND-fed group, ^†^ *P* < 0.05 vs. HFD-fed group.

**CA1 area:** In the ND-sham group, weak 4-HNE immunoreactivity was observed in CA1 pyramidal cells (Figure 6Aa). In the ND-ischemia group, 4-HNE immunoreactivity in CA1 pyramidal cells was significantly increased at 1 day and 2 days after tGCI by about 72% and 61%, respectively, compared to the ND-sham group (Figure 6Ab, Ac, C). At 5 days after tGCI, 4-HNE immunoreactivity was hardly observed in the pyramidal cells due to the loss of CA1 pyramidal cells (Figure 6Ad, C). In the HFD-sham group, 4-HNE immunoreactivity in CA1 pyramidal cells was increased by about 85% compared to the ND-sham group (Figure 6Ae, C). In the HFD-ischemia group, 4-HNE immunoreactivity was more increased (about 120% of the HFD-sham group) at 1 day after tGCI; however, at 2 days and 5 days after tGCI, 4-HNE immunoreactivity was hardly shown in CA1 pyramidal cells due to their death (Figure 6Af, Ag, Ah, C). In the HFD/Fucoidan-sham and ischemia groups, 4-HNE immunoreactivity in CA1 pyramidal cells was not different from that in the ND-sham and ischemia groups (Figure 6Ai, Aj, Ak, Al, C). In addition, we found that change pattern in 4-HNE protein levels in the CA1 area of the ND-, HFD-and HFD/Fucoidan-fed groups was similar to the result of immunohistochemistry (Figure 7A).

**CA2/3 area:** 4-HNE immunoreactivity in CA1 pyramidal cells of the ND-sham group was weakly detected (Figure 6Ba′). In the ND-ischemia group, 4-HNE immunoreactivity in CA1 pyramidal cells was increased at 1 day after tGCI by about 161%, and the increased 4-HNE immunoreactivity was maintained until 5 days after tGCI (Figure 6Bb′–Bd′, D). In the HFD-sham group, 4-HNE immunoreactivity in CA2/3 pyramidal cells was about 171% of the ND-sham group (Figure 6Be′, D). In the HFD-ischemia group, 4-HNE immunoreactivity in CA2/3 pyramidal cells was increased at 1 day and 2 days after tGCI by about 41% and 35%, respectively, compared to the HFD-sham group (Figure 6Bf′, Bg′, D). At 5 days after tGCI, 4-HNE immunoreactivity was very low due to the loss of CA2/3 pyramidal cells (Figure 6Bh′, D). In the HFD/Fucoidan-sham and ischemia groups, 4-HNE immunoreactivity in CA2/3 pyramidal cells was similar to that in the ND-sham and ischemia groups (Figure 6Bi′–Bl′, D). In western blot analysis for changes in 4-HNE protein levels in the CA2/3 area of the ND-, HFD-and HFD/Fucoidan-fed groups, the patterns were similar to those observed in the immunohistochemical data (Figure 7B).

This result suggests that pretreated fucoidan inhibits the obesity-induced increase of lipid peroxidation (4-HNE) in the CA1 and CA2/3 areas in pre- and post-ischemic phase.

### 2.5. Enhancement of Antioxidants (SODs) by Fucoidan in HFD-Ischemia Group

**Figure 8:**
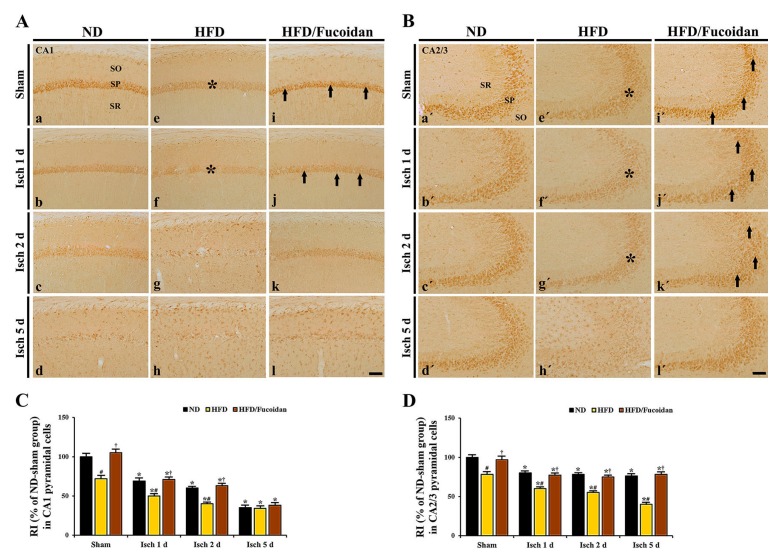
(**A**,**B**) SOD1 immunohistochemistry in the CA1 (**A**) and CA2/3 (**B**) areas of the ND-fed (left columns), HFD-fed (middle columns) and HFD/Fucoidan-fed (right columns) groups at sham (a, e i, a′, e′, i′), 1 day (b, f, j, b′, f′, j′), 2 days (c, g, k, c′, g′, k′) and 5 days (d, h, l, d′, h′, l′) after tGCI. In the HFD-fed group, SOD1 immunoreactivity in CA1-3 pyramidal cells (asterisks) is significantly lower than that in the ND-fed group. However, in the HFD/Fucoidan-fed group, SOD1 immunoreactivity in CA1-3 pyramidal cells (arrows) is significantly increased compared to the HFD-fed group. Scale bar = 60 μm. (**C**,**D**) Quantitative analysis of SOD1 immunoreactivity in the CA1 (**C**) and CA2/3 (**D**) pyramidal cells (*n* = 7/group). The bars indicate the means ± SEM. * *P* < 0.05 vs. each sham group, ^#^ *P* < 0.05 vs. ND-fed group, ^†^ *P* < 0.05 vs. HFD-fed group.

**Figure 9:**
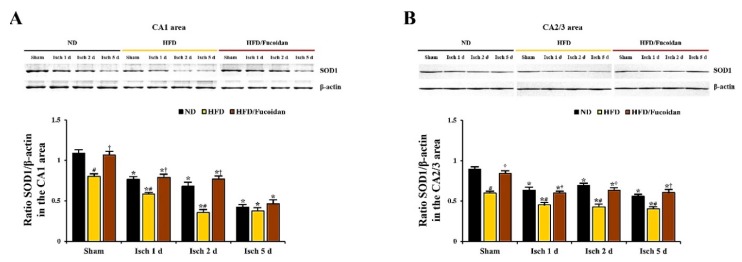
Western blot analysis of SOD1 in the CA1 (**A**) and CA2/3 (**B**) areas (*n* = 6/group). Protein expression is normalized to β-actin. The bars indicate the means ± SEM. * *P* < 0.05 vs. each sham group, ^#^ *P* < 0.05 vs. ND-fed group, ^†^ *P* < 0.05 vs. HFD-fed group.

**Figure 10:**
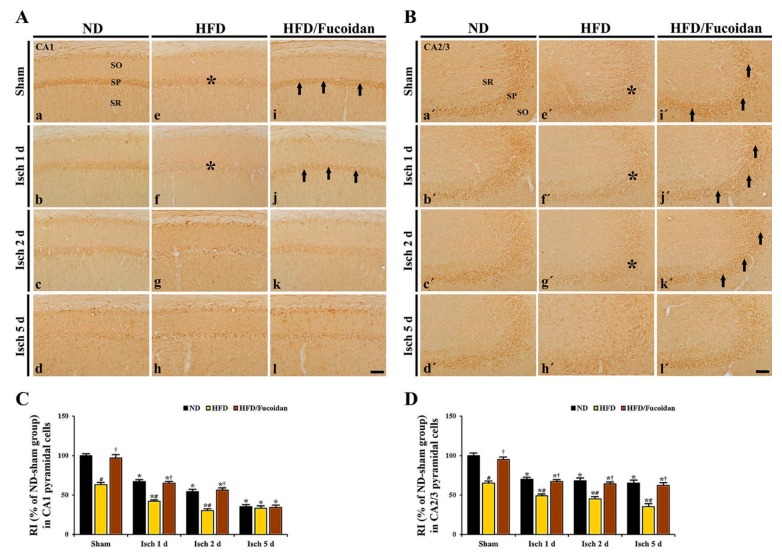
(**A**,**B**) SOD2 immunohistochemistry in the CA1 (**A**) and CA2/3 (**B**) areas of the ND-fed (left columns), HFD-fed (middle columns) and HFD/Fucoidan-fed (right columns) groups at sham (a, e i, a′, e′, i′), 1 day (b, f, j, b′, f′, j′), 2 days (c, g, k, c′, g′, k′) and 5 days (d, h, l, d′, h′, l′) after tGCI. Asterisks indicate that the SOD2 immunoreactivity in CA1-3 pyramidal cells of the HFD-fed group is significantly decreased. However, SOD2 immunoreactivity in CA1-3 pyramidal cells of the HFD/Fucoidan-fed group (arrows) is similar to the ND-fed group. Scale bar = 60 μm. (**C**,**D**) Quantitative analysis of SOD2 immunoreactivity in the CA1 (**C**) and CA2/3 (**D**) pyramidal cells (*n* = 7/group). The bars indicate the means ± SEM. * *P* < 0.05 vs. each sham group, ^#^ *P* < 0.05 vs. ND-fed group, ^†^ *P* < 0.05 vs. HFD-fed group.

**Figure 11:**
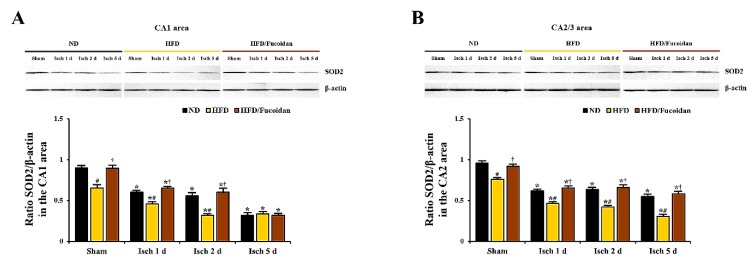
Western blot analysis of SOD2 in the CA1 (**A**) and CA2/3 (**B**) areas (*n* = 7/group). Protein expression is normalized to β-actin. The bars indicate the means ± SEM. * *P* < 0.05 vs. each sham group, ^#^ *P* < 0.05 vs. ND-fed group, ^†^ *P* < 0.05 vs. HFD-fed group.

**CA1 area:** In the ND-sham group, strong SOD1 and SOD2 immunoreactivity was observed in CA1 pyramidal cells (Figure 8Aa and Figure 10Aa). In the ND-ischemia group, SOD1 and SOD2 immunoreactivity in CA1 pyramidal cells was significantly decreased to about 69% and 67%, respectively, of the ND-sham group at 1 day after tGCI (Figure 8Ab, C and Figure 10Ab, C), and, at 2 days after tGCI, SOD1 and SOD2 immunoreactivity was more decreased (Figure 8Ac, C and Figure 10Ac, C). 5 days after tGCI, SOD1 and SOD2 immunoreactivity in CA1 pyramidal cells was very low due to the death of them (Figure 8Ad, C and Figure 10Ad, C). In the HFD-sham group, SOD1 and SOD2 immunoreactivity in CA1 pyramidal cells was significantly lower (about 72% and 78% of the ND-sham group, respectively) than that of the ND-sham group (Figure 8Ae, C and Figure 10Ae, C). In the HFD-ischemia group, SOD1 and SOD2 immunoreactivity in CA1 pyramidal cells was significantly decreased from 1 day after tGCI, and, at 5 days after tGCI, SOD1 and SOD2 immunoreactivity was rarely observed in CA1 pyramidal cells due to their death (Figure 8Af–Ah, C and Figure 10Af–Ah, C). In the HFD/Fucoidan-sham group, SOD1 and SOD2 immunoreactivity in CA1 pyramidal cells was significantly higher (about 146% and 153% of the HFD-sham group, respectively) than that of the HFD-sham group (Figure 8Ai, C and Figure 10Ai, C). In the HFD/Fucoidan-ischemia group, SOD1 and SOD2 immunoreactivity in CA1 pyramidal cells was decreased with time after tGCI, but each immunoreactivity was higher than that in the HFD-ischemia group (Figure 8Aj–Al, C and Figure 10Aj–Al, C). In addition, we found that change patterns in SOD1 and SOD2 protein levels in the CA1 area of all groups were similar to the results of their immunohistochemistry (Figure 9A and Figure 11A).

**CA2/3 area:** In the ND-sham group, SOD1 and SOD2 immunoreactivity in CA2/3 pyramidal cells was similar to that in CA1 pyramidal cells (Figure 8Ba′ and Figure 10Ba′). In the ND-ischemia group, SOD1 and SOD2 immunoreactivity in CA2/3 pyramidal cells was significantly decreased to about 79% and 70%, respectively, of the ND-sham group at 1 day after tGCI, and their immunoreactivity was not significantly changed until 5 days post-tGCI (Figure 8Bb′–Bd′, D and Figure 10Bb′–d′, D). In the HFD-sham group, SOD1 and SOD2 immunoreactivity in CA2/3 pyramidal cells was significantly lower (about 78% and 66% of the ND-sham group, respectively) than that in the ND-sham group (Figure 8Be′, D and Figure 10Be′, D). In the HFD-ischemia group, SOD1 and SOD2 immunoreactivity was gradually decreased with time, and, at 5 days after tGCI, SOD1 and SOD2 immunoreactivity was very low due to the damage of CA2/3 pyramidal cells (Figure 8Bf′–Bh′, D and Figure 10Bf′–Bh′, D). In the HFD/Fucoidan-sham and ischemia groups, SOD1 and SOD2 immunoreactivity was similar to that in the ND-fed group (Figure 8Bi′–Bl′, D and Figure 10Bi′–Bl′, D). Also, western blot analysis showed that change patterns in SOD1 and SOD2 protein levels in the CA2/3 in all groups were similar to those observed in their immunohistochemical data (Figure 9B and Figure 11B).

This finding indicates that pretreated fucoidan increases the obesity-mediated decreases of levels of antioxidant enzymes (SODs) in the CA1 and CA2/3 areas in pre- and post-ischemic phases.

## 3. Discussion

Numerous candidate neuroprotective agents for brain ischemia have been identified in experimental studies, however, most of them have failed to confer neuroprotection in clinical trials [30]. A crucial reason is that most animals used in experimental studies do not mimic the clinical state of patients with brain ischemia who generally have comorbidities, such as diabetes, hypertension, and obesity [31,32]. Accordingly, it is necessary to test candidate agents against ischemic brain injury not only in normal animals but also in animals with comorbidities. First of all, in this study, we set up an animal model of HFD-induced obesity to investigate neuroprotective effects of fucoidan against ischemic brain injury in animals with comorbidities, showing that the gerbils fed HFD for 12 weeks showed significant increases in body weight, blood glucose, serum triglyceride and total cholesterol levels. These results are consistent with results of a previous study [16].

Accumulating evidence exists to suggest that HFD-induced obesity and related metabolic abnormalities result in the exacerbation of transient ischemia-induced brain injury. For example, it was reported that obesity induced by HFD increased cerebral infarction with severe neurological deficits in rat and mouse models of transient focal cerebral ischemia [33,34]. In addition, we recently reported that HFD-induced hyperglycemia and hyperlipidemia led to the augmentation of ischemic brain injury in the septum and hippocampus in gerbils submitted to tGCI [35,36]. In accordance with the above-mentioned previous studies, our current study showed that CA1 pyramidal cell death in the HFD-fed obese gerbils occurred at 2 days after tGCI compared to that in the ND-fed non-obese gerbils, and massive neuronal death in the HFD-fed obese gerbils was observed in the CA2/3 area, which is relatively resistant to tGCI than the CA1 area [37,38], at 5 days after tGCI, indicating that HFD-induced obesity could elicit the acceleration and exacerbation of tGCI-induced neuronal death in the CA1 and CA2/3 areas. Interestingly, pretreated fucoidan in the HFD-fed obese gerbils significantly alleviated pyramidal cell death in the CA1 and CA2/3 areas at 2 days and 5 days, respectively, after tGCI without affecting blood glucose and serum lipid concentrations. These results indicate that fucoidan could afford neuroprotection against obesity-induced augmentation of ischemic brain damage.

A large body of evidence suggests that ROS-mediated oxidative stress leads to damage to macromolecules, including DNA, proteins, and lipids, which are directly or indirectly related to neuronal death induced by transient ischemia [39,40]. In addition, it has been reported that HFD-induced obesity is accompanied by an increase of oxidative stress in the brain, which results in brain dysfunction [11,41]. In this respect, we, in this study, found that levels of DHE (a selective probe for superoxide anion), 8-OHG (a marker of hydroxyl radical damage to RNA) and 4-HNE (an end-product of lipid peroxidation) as indicators of oxidative stress were significantly increased in the CA1 and CA2/3 areas of the HFD-sham group compared to those in the ND-sham group. In addition, DHE, 8-OHG, and 4-HNE levels in the HFD-fed obese gerbils were significantly increased in the CA1 and CA2/3 areas at 1 day and 2 days, respectively, after tGCI (before the times when the tGCI-induced death of CA1 and CA2/3 pyramidal cells occurs), compared to the ND-fed non-obese gerbils. These findings indicate that oxidative stress must be further enhanced in HFD-fed obese animals at an early time after tGCI and that, furthermore, these levels are significantly decreased by pretreatment with fucoidan.

Enzymatic antioxidant defense systems play important roles in protecting neurons from transient ischemia-induced brain oxidative stresses [9,42]. Among antioxidant enzymes, the protective role of SODs has extensively been studied in in vivo models of brain ischemia because SODs constitute the first line of defense against ROS. In this context, some researchers have demonstrated that a deficiency of SOD1 or SOD2 in mutant mice results in the exacerbation of brain injury following transient focal and global cerebral ischemia via increased oxidative stress [43,44]. On the other hand, it has been studied that ischemic brain damage after transient global or focal cerebral ischemia is significantly attenuated in transgenic mice and rats overexpressing SOD1 or SOD2 [45,46,47]. Anyway, however, many studies have indicated that increased levels of endogenous SODs by pharmaceutical administration may contribute to protection against ischemic brain injury [48,49,50]. Although there is no information on the antioxidant effects of fucoidan against tGCI-induced brain oxidative damage in experimental models of HFD-induced obese or metabolic disorders, a previous study has shown that pretreatment with fucoidan attenuates oxidative stress through decreasing ROS concentrations and restoring SOD activity in PC_12_ cells caused by H_2_O_2_-induced injury [51]. Furthermore, we recently reported that pretreated fucoidan effectively alleviated tGCI-induced oxidative stress in the hippocampal CA1 area of non-obese gerbils via reducing lipid peroxidation and superoxide anion radical generation as well as increasing expression levels of endogenous SODs [23]. In this study, we found that expression levels of SOD1 and SOD2 in the CA1 and CA2/3 areas of the HFD-fed obese gerbils at sham, 1 day and 2 days after tGCI were significantly lower than those in the ND-fed obese gerbils. In addition, the HFD/fucoidan-fed obese gerbils showed significant increase in the obesity-mediated decreases of levels of SOD1 and SOD2 in the pre- and post-ischemic phases. Consequently, based on the previous studies and our present findings, we suggest that antioxidant properties of fucoidan is still evident in HFD-fed obese animals, which may contribute to fucoidan-mediated attenuation of the acceleration and exacerbation of tGCI-induced injury. However, weaknesses of this study include the lack of data on enzymatic and molecular experiments, such as expressions of genes, which should be obtained in future studies.

In conclusion, results of this study clearly show that pretreated fucoidan could alleviate the acceleration and exacerbation of tGCI-induced death of CA1 and CA2/3 pyramidal cells in HFD-fed obese gerbils, and the alleviation might be closely associated with the attenuation of obesity-induced severe oxidative stress in pre- and post-ischemic phases by fucoidan pretreatment. Taken together, our findings strongly suggest that fucoidan has excellent potential as a candidate agent in attenuating severe ischemic brain injury in obese patients who have high cerebral ischemic risk.

## 4. Materials and Methods

### 4.1. Experimental Animals

Male Mongolian gerbils were used at six months (body weight, 72–78 g) of age. They were housed under a 12 h light/12 h dark cycle, constant temperature (22–23 °C) and relative humidity (55-60%), and they were allowed free access to water and food. All experimental procedures were performed in accordance with the guidelines that are in compliance with the current international laws and policies (Guide for the Care and Use of Laboratory Animals, The National Academies Press, 8th Ed., 2011) and approved (approval no., KW-180124-1, approval date: 22 May 2018) by the Institutional Animal Care and Use Committee (IACUC) at Kangwon National University (Chuncheon, Republic of Korea).

### 4.2. Experimental Design and Fucoidan Administration

Gerbils were randomly divided into 6 groups: (1) gerbils fed ND and received sham tGCI (ND-sham group, *n* = 13), (2) gerbils fed ND and received tGCI (ND-ischemia group, *n* = 39), (3) gerbils fed HFD and sham tGCI (HFD-sham group, *n* = 13), (4) gerbils fed HFD and received tGCI (HFD-ischemia group, *n* = 39), (5) gerbils fed HFD plus fucoidan and received sham tGCI (HFD/Fucoidan-sham-group, *n* = 13), and (6) gerbils fed HFD plus fucoidan and received tGCI (HFD/Fucoidan-ischemia group, *n* = 39).

The gerbils were fed a commercially available rodent diet, which consisted of different fat concentrations as follows: ND (D12450B, 10% kcal% fat, 20% kcal% protein, 70 % kcal% carbohydrate, Research Diets, NJ, USA) or HFD (D12492, 60% kcal% fat, 20% kcal% protein, 20% kcal% carbohydrate, Research Diets). All gerbils were allowed free access to water and food for 12 weeks.

**Figure 12:**
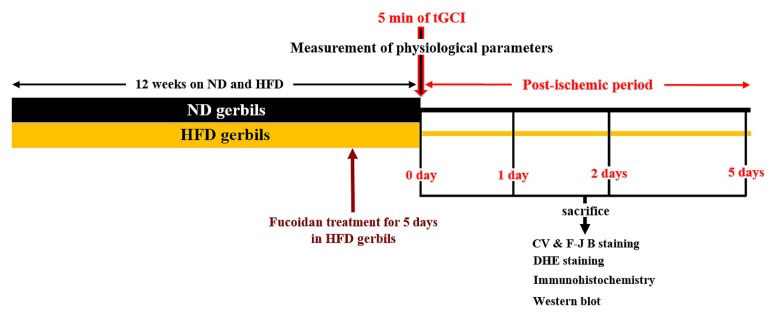
Experimental design. ND or HFD was administered for 12 weeks, and fucoidan was administered to the HFD-fed gerbils for the last 5 days. 12 weeks after the feeding, physiological parameters (body weight, blood glucose levels, and serum lipid concentrations) were measured. After the measurement, the gerbils were subjected to 5 min of tGCI and sacrificed at sham, 1 day, 2 days, and 5 days after tGCI for various analyses.

Fifty mg/kg of fucoidan (Sigma-Aldrich, Poole, Dorset, UK) extracted from *Fucus vesiculosus* was dissolved in sterile saline and injected intraperitoneally once a day for the last 5 days during HFD exposure as shown in Figure 12. The dosage (50 mg/kg) of fucoidan was selected based on our published report, in which 50 mg/kg fucoidan displayed strong neuroprotection against tGCI-induced neuronal injury in normal animals [23].

### 4.3. Analysis of Body Weight, Glucose Level and Lipid Profiles

Body weight was measured weekly. Blood glucose and serum lipid profiles were measured at 12 weeks after feeding according to our published method [16]. In brief, the animals were anesthetized with pentobarbital sodium (60 mg/kg, i.p.) (JW Pharmaceutical, Seoul, Korea). Blood sample was collected from each animal by orbital puncture, and blood glucose level was analyzed by using a blood glucose monitor (Ascensia Elite XL Blood Glucose Meter, Bayer, Toronto, ON, Canada). In addition, serum was separated from the blood by centrifugation at 13,000 *g* for 25 min at 4 °C (centrifuge 5424R, Eppendorf, Hamburg, Germany) and stored at −80 °C until analysis. Total cholesterol and triglyceride level in the serum was measured enzymatically by using a dry chemistry analyzer (FUJI DRI-CHEM NX500; Fujifilm, Japan).

### 4.4. Induction of tGCI

tGCI was induced according to our published method [16]. In short, the gerbils were anesthetized with a mixture of 2.5% isoflurane in 34% oxygen and 66% nitrous oxide. Bilateral common carotid artery occlusion (BCCAO) was done for 5 min, and the restoration of blood flow (reperfusion) was directly observed under an ophthalmoscope (HEINE K180, Heine Optotechnik, Herrsching, Germany). Body (rectal) temperature was maintained under normothermic condition (37 ± 0.5 °C). For the sham-tGCI operation, the same surgical procedure was carried out, except for BCCAO.

### 4.5. Preparation of Histological Sections

As we described previously [16], the gerbils (*n* = 7 at sham, 1 day, 2 days and 5 days after BCCAO) were anesthetized with pentobarbital sodium (60 mg/kg, i.p.) (JW Pharmaceutical), and they were fixed transcardially with 4% paraformaldehyde. Their brains were removed and fixed in the same fixative for 8 h and infiltrated with 30% sucrose for cryoprotection overnight. Finally, serial 30-μm coronal sections were made in a cryostat (Leica, Wetzlar, Germany).

### 4.6. Cresyl Violet (CV) Staining

To examine the change of cell distribution in the hippocampus after tGCI, the sections of each group were stained with CV. As we descried previously [3], in short, the sections were stained with CV acetate (Sigma, St. Louis, MO, USA) solution (1.0% (*w*/*v*)) for 2 min at room temperature. The stained sections were washed twice and dehydrated by immersing them in ethanol baths (50%, 70%, 80%, 90%, 95% and 100%) at room temperature.

### 4.7. Fluoro-Jade B (F-J B) Histofluorescence Staining

To investigate cell damage/death in the hippocampus after tGCI, F-J B (Histochem, Jefferson, AR, USA) histofluorescence staining was done according to a published procedure [16]. In brief, the sections were immersed in 1% sodium hydroxide solution, transferred to 0.06% potassium permanganate solution and reacted with 0.0004% Fluoro-Jade B solution on a slide warmer (approximately 50 °C).

### 4.8. Dihydroethidium (DHE) Fluorescence Staining

To evaluate in the situ production of superoxide anion, oxidative fluorescent dye DHE (Sigma-Aldrich) was used. The detection of superoxide anion radical was performed as previously described [52]. In brief, the sections were equilibrated in Krebs-HEPES buffer (130 mM NaCl, 2 mM CaCl2, 5.6 mM KCl, 0.24 mM MgCl2, 8.3 mM HEPES, 11 mM glucose) (pH 7.4) for 30 min at 37 °C. Fresh buffer containing DHE (10 μmol/L) was applied to the sections. The sections were coverslipped and incubated in a light-protected humidified chamber for 2 h at 37 °C.

### 4.9. Immunohistochemistry

Immunohistochemistry was performed to examine (1) neuronal damage by using neuron-specific soluble nuclear antigen (NeuN, a marker for neurons), (2) oxidative stress by using 8-hydroxyguanine (8-OHG, a marker of RNA oxidative degradation) and 4-hydroxy-2-nonenal (4-HNE, a marker for lipid peroxidation), (3) endogenous antioxidants by using SOD1 and SOD2 as primary antibodies. Immunohistochemistry for each antibody was done according to our published procedure [52]. In brief, the sections were incubated with each diluted antibody as follows: mouse anti-NeuN (1:1000, Chemicon, Temecula, CA, USA), goat anti-8-OHG (1:500, Thermo Scientific, Waltham MA, USA), mouse anti-HNE (1:1,000, Alexis Biochemicals, San Diego, CA, USA), sheep anti-SOD1 (1:1000, Calbiochem, La Jolla, CA, USA) or sheep anti-SOD2 (1:1000, Calbiochem). Continuously, the incubated sections were exposed to biotinylated rabbit anti-mouse, goat or sheep IgG (1:250, Vector Laboratories Inc., Burlingame, CA, USA) and streptavidin peroxidase complex (1:250, Vector). Finally, the reacted sections were visualized by using 3,3′-diaminobenzidine (Sigma-Aldrich).

For establishing the specificity of each immunostaining, each negative control test was done with preimmune serum instead of each primary antibody. The test showed no immunostained structures in the sections as previously described [16].

### 4.10. Western Blot Analysis

To assess levels of 4-HNE, SOD1 and SOD2 in the CA1 or CA2/3 area following tGCI, animals (*n* = 6 at sham, 1 day, 2 days, and 5 days after tGCI) were used for western blot analysis according to our published method [53]. In short, the CA1 or CA2/3 tissues were homogenized in 50 mM PBS (pH 7.4) containing ethylene Glycol tetraacetic acid (pH 8.0), 10 mM ethylenediaminetetraacetic acid (pH 8.0), 0.2% Nonidet P-40, 15 mM sodium pyrophosphate, 100 mM β-Glycerophosphate, 50 mM sodium fluoride, 150 mM sodium chloride, 2 mM sodium orthvanadate, 1 mM phenylmethylsulfonyl fluoride and 1 mM dithiothreitol (DTT). The homogenized tissues were centrifuged at 14,000 × *g* for 35 min at 4 °C. Protein levels in the supernatants were determined by using a Micro bicinchoninic acid Protein Assay kit with bovine serum albumin (Pierce Chemical, Rockford, IL, USA). The aliquots containing 50 µg total protein were boiled in a loading buffer containing 250 mM Tris (pH 6.8), 10 mM DTT, 10% sodium dodecyl sulfate, 0.5% bromophenol blue and 50% Glycerol, and they were subsequently loaded onto a 10% polyacrylamide gel (Sigma-Aldrich). After the electrophoresis, the gels were transferred onto nitrocellulose membranes (Pall Corp., Pittsburgh, PA, USA). The membranes were subsequently incubated with diluted mouse anti-HNE (1:1000, Alexis Biochemicals), sheep anti-SOD1 (1:1000, Calbiochem), sheep anti-SOD2 (1:500, Calbiochem) and mouse anti-β-actin (1:5000, Sigma-Aldrich) overnight at 4 °C. Finally, they were exposed to peroxidase conjugated goat anti-mouse or sheep IgG (1:4000, Santa Cruz Biotechnology, Santa Cruz, CA, USA) and an enhanced chemiluminescence kit (GE Healthcare Life Sciences, Chalfont, UK).

### 4.11. Data Analysis

To quantitatively analyze neuronal death, three sections/animal were selected with 120 μm interval (anteroposterior −1.4 to −2.2 mm of the gerbil brain atlas) [54]. NeuN-immunoreactive (^+^) and F-J B-positive (^+^) cells were counted as previously described [55]. In short, digital images of NeuN^+^ and F-J B^+^ cells were obtained under a light microscope (BX53, Olympus, Hamburg, Germany) and an epifluorescent microscope (Carl Zeiss, Göttingen, Germany) with blue (450–490 nm) excitation light and a barrier filter, respectively. The cells were counted in a 250 × 250 μm square at the center of the CA1 or CA2/3 area. Cell counts were obtained by averaging total numbers by using an image analyzing system (software: Optimas 6.5, CyberMetrics, Scottsdale, AZ, USA).

The fluorescence intensity of DHE immunoreaction was measured according to our published method [52]. In brief, digital images were captured from the CA1 or CA2/3 area under an epifluorescence microscope (Olympus BX53) with an excitation wavelength of 520–540 nm, and the DHE fluorescence intensity was analyzed by using Image-pro Plus 6.0 software. A ratio of the DHE fluorescence intensity was calibrated as %, with the ND-sham group designated as 100%.

The density of 8-OHG^+^, 4-HNE^+^, SOD1^+^ and SOD2^+^ structure was quantitatively analyzed according to our published method [3]. In brief, a digital image of each immunoreactive structure was taken like the above-mentioned method. Each image was calibrated into an array of 512 × 512 pixels. The immunoreactivity of each structure was evaluated on the basis of an optical density (OD), which was obtained after transformation of the mean gray level using the formula: OD = log (256/mean gray level). Finally, we compared them as a ratio of relative immunoreactivity (RI) for each immunoreactive structure: RI was calibrated as % by using Adobe Photoshop (version 8.0, San Jose, CA, USA) and NIH Image software (1.59). A ratio of the RI was calibrated as %, with the ND-sham group designated as 100%.

Results of the western blotting were analyzed according to our published procedure [16]. Briefly, bands of 4-HNE, SOD1 and SOD2 obtained from the CA1 or CA2/3 area were scanned by using a ChemiDoc Imaging System (Bio-Rad Laboratories, Inc., Hercules, CA, USA), and densitometric analysis for the quantification of the western bands was done by using Scion Image software (Scion Corp., Frederick, MD, USA). The expression rate of the target protein was normalized through the corresponding expression rate of β-actin.

### 4.12. Statistical Analysis

Data are presented as means ± standard errors of the mean (SEM). All statistical analyses were performed by using GraphPad Prism (version 5.0; GraphPad Software, La Jolla, CA, USA). A multiple-sample comparison was applied to test the tGCI-related differences between the groups (two-way analysis of variance [ANOVA] and the Bonferroni’s multiple comparison test as post hoc test using the criterion of the least significant differences). Statistical significance was considered at *P* < 0.05.

## Figures and Tables

**Table 1:** Changes in body weight, blood glucose levels, and serum lipid concentrations in the ND-fed group, HFD-fed group and HFD/Fucoidan-fed group.

Parameters	ND	HFD	HFD/Fucoidan
Body weight (g)	81.2 ± 0.7	112.5 ± 1.3 *	105.7 ± 0.5 *
Glucose (mg/dL)	95.5 ± 2.5	181.2 ± 3.7 *	170.1 ± 2.9 *
Triglyceride (mg/dL)	91.6 ± 3.8	169.3 ± 4.2 *	162.7 ± 3.5 *
Total cholesterol (mg/dL)	101.4 ± 4.2	185.9 ± 5.1 *	179.8 ± 6.2 *

Values are expressed as mean ± SEM (*n* = 13 gerbils per group). ND, normal diet; HFD, high-fat diet. * *P* < 0.05 vs. ND-fed group.
